# Relationship between the Performance in a Maximum Effort Test for Lifeguards and the Time Spent in a Water Rescue

**DOI:** 10.3390/ijerph18073407

**Published:** 2021-03-25

**Authors:** Sergio López-García, Brais Ruibal-Lista, José Palacios-Aguilar, Miguel Santiago-Alonso, José Antonio Prieto

**Affiliations:** 1 Faculty of Education, Pontifical University of Salamanca, 37003 Salamanca, Spain; slopezga@upsa.es; 2 Group of Investigation in Preventive and Lifesaving Activities (GIAPS), University of A Coruña, 15179 A Coruña, Spain; palacios@udc.es; 3 Faculty of Sport Sciences and Physical Activity, University of A Coruña, 15179 A Coruña, Spain; miguel.santiago@udc.es; 4 Faculty Padre Ossó, University of Oviedo, 33008 Oviedo, Spain; josea@facultadpadreosso.es

**Keywords:** water rescue, physical fitness, lifesaving, vo2max, performance, drowning

## Abstract

The main objective of this study was to analyse the relationship between the performance in a maximum incremental test for lifeguards, the IPTL, and the effectiveness of a 200 m water rescue on the beach. Initially, 20 professional lifeguards carried out the IPTL in the pool and then they performed a 200 m water rescue on the beach. The maximum oxygen uptake (VO_2max_) in the IPTL was estimated. In both tests, heart rate (HR), blood lactate (La) and time achieved were measured. The VO_2max_ estimated in the IPTL (VO_2IPTL_) was 44.2 ± 4.7 mL·kg·min^−1^, the time reached in the IPTL (Time_IPTL_) was 726 ± 72 s and the time spent in the rescue (Time_Rescue_) was 222 ± 14 s. The results showed that the time reached in the pool (Time_IPTL_) was the best predictor variable of the performance in water rescue (Time_Rescue_) (R^2^ = 0.59; *p* < 0.01). A significant correlation was also observed between the estimated maximum oxygen uptake and the beach rescue performance (R^2^ = 0.37; *p* = 0.05). These results reveal that the IPTL, a maximum incremental test specific to lifeguards, allows the estimation of the effectiveness of a 200 m rescue on the beach.

## 1. Introduction

Drowning is one of the highest causes of accidental death in the world. According to the World Human Organization (WHO), 372,000 people die from drowning every year worldwide [1].

In the process of drowning by submersion, a person can go into cardiac arrest in less than two minutes [2,3]. In cases where victims initially survive the drowning incident, quickly reversing hypoxia is key to preventing cardiac arrest [4]. However, when a cardiac arrest is suffered, it is vitally important to start the Basic Life Support (BLS) manoeuvres as soon as possible, since the rapid intervention with the BLS is associated with a higher probability of survival [5]. Consequently, early intervention in the event of a drowning by performing a water rescue is necessary.

One of the most important factors to take into consideration when performing a water rescue is the physical condition of lifeguards, determined by the physiological demands of the rescue. In this sense, it has been shown that the values of heart rate [6,7], blood lactate [8,9] and oxygen uptake [10] increase until they approach maximum values during a rescue, which means that aerobic capacity and power are fundamental to the lifeguard’s performance during the rescue [7,11].

With regard to the functional evaluation of the professional lifeguard, there are few tests or protocols included in the scientific literature. In this respect, a specific maximum effort test, the Incremental Pool Test for Lifeguards (IPTL), has recently been designed and validated, which allows for the estimation of the lifeguard’s maximum oxygen uptake (VO_2_max) in a simple and reliable way [12]. The association between IPTL and the performance of a water rescue has not been proved yet; however, doing so could provide more information about the physical demands of the latter, and improve lifeguard training.

This research aims to analyse the association between IPTL performance and the effectiveness of a simulated 200 m water rescue on the beach.

## 2. Materials and Methods

### 2.1. Sample

Twenty-five subjects participated, 22 men and 3 women, of whom 20 men completed the two tests carried out. All the participants signed an informed consent form once the research methodology and the purpose of the tests were explained.

This research was developed under the ethical principles of the Declaration of Helsinki for biomedical studies with human beings and the whole study was supported and accepted by the research and teaching ethics committee of the University of A Coruña (CEI-UDC).

### 2.2. Study Tests

First, medical and anthropometric assessments of all the participants were carried out.

#### 2.2.1. Anthropometric Test

Height, weight, body mass index and percentage of fat mass and muscle mass were analysed during the anthropometric test. Height and weight were measured with the Astra GIMA scale, with the participant in a standing position, looking straight and without shoes.

The protocol to measure the percentages of fat and muscle mass was based on the one proposed by Norton and Eston [13], with the measurement of six skin folds (tricipital, subscapular, supraspinal, abdominal, anterior thigh and medial leg) and three bone diameters (bistyloid radius, biepicondyle humerus and bicondylar femur). All measurements were taken from the right side of the body, giving the mean of three measurements as a valid value.

#### 2.2.2. Incremental Pool Test for Lifeguards (IPTL)

The protocol described by Ruibal-Lista et al. [14] was used to carry out the IPTL test. It was conducted in a heated swimming pool of 25 m long, 2.5 m wide and 2 m deep. The IPTL protocol consisted of repeatedly swimming a distance of 25 m at a pre-established and progressive pace using an acoustic sound alert at both ends of the pool. Each intensity level of the test increased progressively after completing 4 repetitions of 25 m (100 m) until the participant was no longer able to maintain the test pace. The maximum time of the test was defined as the time at which the last series of 25 m was completed within the established pace [14]. All lifeguards had flippers to carry out the test and a hi-fi system with amplifier was available to play the acoustic warnings of the test.

#### 2.2.3. Water Rescue

The water rescue was undertaken at Oza beach (A Coruña, Spain), with calm sea (waves less than 0.5 m) and light wind (less than 3 m/s). A rescue was designed 100 m from the shore, similar to that of the study by Kalén et al. (2017) [9]. The rescue protocol consisted of: running 10 m into the water, 100 m of approach swimming with fins, 100 m of towing swim with a mannequin pretending to be an unconscious victim and 10 m of extraction of the mannequin from the water, similar to that of the study by Barcala-Furelos et al. [15].

### 2.3. Variables Analysed

During the anthropometric assessment, the height, weight, fat percentage and muscle mass were analysed, the latter two on the basis of a skin fold analysis [13], using the Faulkner equation [16] and the equation proposed by De Rose and Guimaraes [17], respectively.

HR at rest (HR_Rest_) was also measured during this process. First, the participant was placed on his or her back for 10 min. After that, HR was measured for 1 min with the Suunto Ambit 3 Run pulsometer (Suunto©, Vantaa, Finland).

During the IPTL and the water rescue, the maximum heart rate (HR) obtained (HR_iptl_ y HR_Rescue_) was measured with the Suunto Ambit 3 Run pulsometer (Suunto©, Vantaa, Finland), and the post-exercise blood lactate levels (La) (La_IPTL_ y La_Rescue_) were measured at 1, 3, 5 and 7 min after recovery using a capillary blood sample (15 μL) with Lactate Pro 2 analyser (Laktate, BUSIMEDIC S.L., Donostia, Spain).

The VO_2max_ reached in the IPTL (VO_2IPTL_) was estimated from the formula described by Ruibal-Lista et al. [1]: VO_2IPTL_ = 0.025 Time_IPTL_(s) + 1.69 muscle_mass_(%) + 0.436 weight(kg) − 79.93. Due to the difficulty of measuring VO_2_ directly within water, we used the Heart Rate Reserve [18], to calculate the percentage of VO_2max_ reached in water rescue (VO_2Rescue_). The Heart Rate Reserve was obtained from the HR_IPTL_, since this test has shown that it allows one to obtain the maximum heart rate in the water environment [14].

Finally, the times obtained in both tests (Time_IPTL_ and Time_Rescue_), were measured using the Casio Sport HS-3V-1RET stopwatch by one researcher.

### 2.4. Statistical Analysis

All the data obtained in both phases of the research were stored and analysed using the SPSS statistical package (version for Windows 21.0).

The performance in a water rescue was estimated from the results obtained in the IPTL. Normality was verified by the Shapiro–Wilk test. For this purpose, a linear regression model was applied with the objective of predicting the time in a water rescue (dependent variable) from the variables measured in the IPTL (independent variables). To choose the variables for the regression model, a stepwise regression procedure was used. To determine the degree of validity of the model, the empirical data assumptions of linearity, independence, normality, homoscedasticity and non-collinearity were analysed among the independent variables. A significance level of *p* < 0.05 was established for all analyses.

## 3. Results

### 3.1. Descriptive Data on Anthropometry and Body Composition

Table 1 shows the data related to anthropometry and body composition.

### 3.2. Results in the IPTL

In the pool test (IPTL), heart rate (HR_IPTL_), lactate levels (LA_IPTL_) and test time (TIME_IPTL_) were measured. In addition, the maximum oxygen uptake reached (VO_2IPTL_) was estimated. Table 2 shows the results obtained.

### 3.3. Results in the Rescue

The average HR (HR_Rescue_) was 172 ± 9 beats·min^−1^, lactate levels (La_Rescue_) were 14.0 ± 2.8 mmol·L^−1^ and the average maximum time spent on the rescue (Time_Rescue_) was 222 ± 14 s, as shown in Table 3.

### 3.4. Comparative Analysis between IPTL and Water Rescue

There are significant differences between the results obtained in the IPTL and the water rescue, the HR values being higher in the IPTL (*p* < 0.001) and those of La being lower (*p* = 0.001) (Table 4).

### 3.5. Estimation of Rescue Time from IPTL Performance

Table 5 shows the correlations observed between the different variables measured in the IPTL and the effectiveness during the water rescue.

The introduction of the variables into the model was carried out through a protocol of successive steps where the time invested in the rescue (Time_Rescue_) was selected as a dependent variable and the variables measured in the IPTL that correlated significantly with that time (Time_IPTL_ and VO_2IPTL_), as independent variables. Only one variable was introduced finally into the model, which was the maximum time reached in the IPTL (Time_IPTL_). The degree of explanation of the model is 59.1% (R^2^) (Table 6).

Table 7 shows that the significance of F in the selected model is less than 0.001, which indicates that the model is suitable to explain the dependent variable. Therefore, it is stated that there is a relationship between the variable X1 (independent) and the variable Y (dependent).

Table 8 shows the coefficients of the model. From these results, the regression equation will take the expression: Time_Rescue_ = 332.98 − 0.154 Time_IPTL_ (s).

Knowledge of the residues provided the necessary information to study the compliance with the assumptions of the regression model.

The assumption of independence of the residues is confirmed when applying Durbin–Watson statistics (since the values obtained are between 1.5 and 2.5 (1.511).

**Figure 1:**
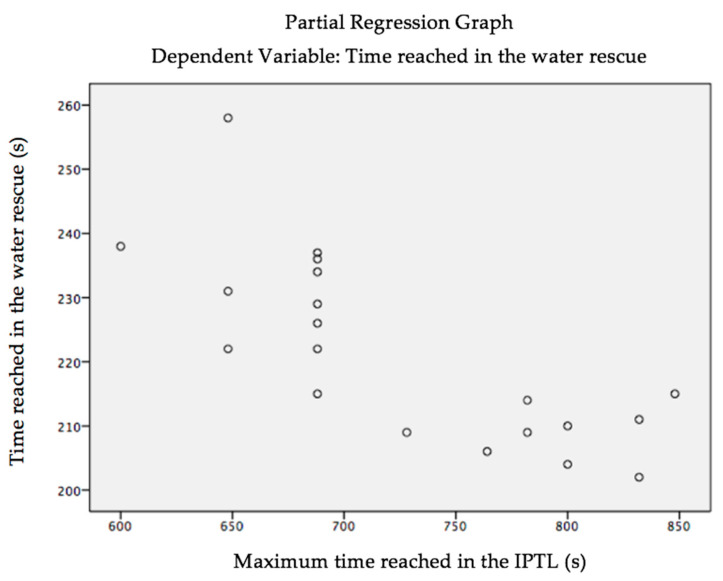
Partial regression between time spent in the rescue (TimeRescue) and time reached in the Incremental Test in Pool for Lifeguards (IPTL) (TimeIPTL).

As can be seen in the graph below, linearity is evident between the dependent variable and the independent variable (Figure 1).

**Figure 2:**
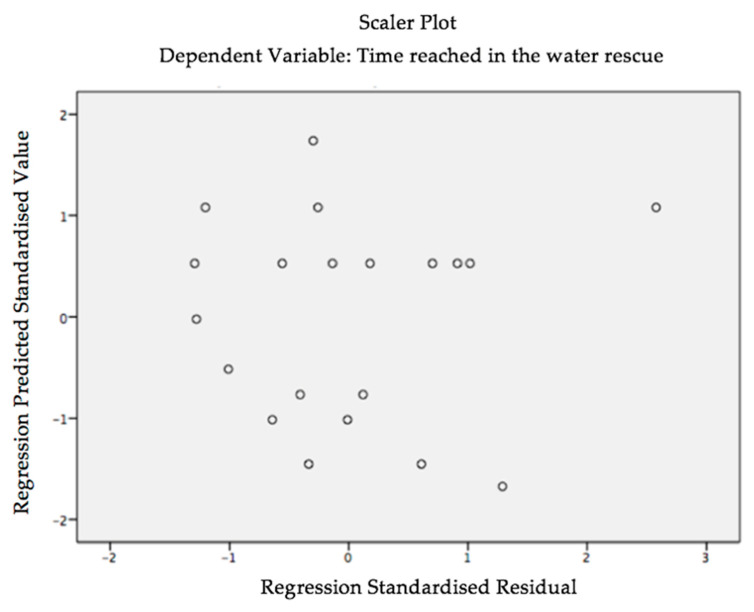
Predicted values according to the standardised residues for the dependent variable (Time_Rescue_).

In the graph of the predicted values according to the standardised residues for the dependent variable (Time_Rescue_), there is no trend in the distribution of residues, so the assumption of homoscedasticity is confirmed, as the residues are randomly distributed in a range between +2.5 and −2.5 standard deviations (Figure 2).

**Figure 3:**
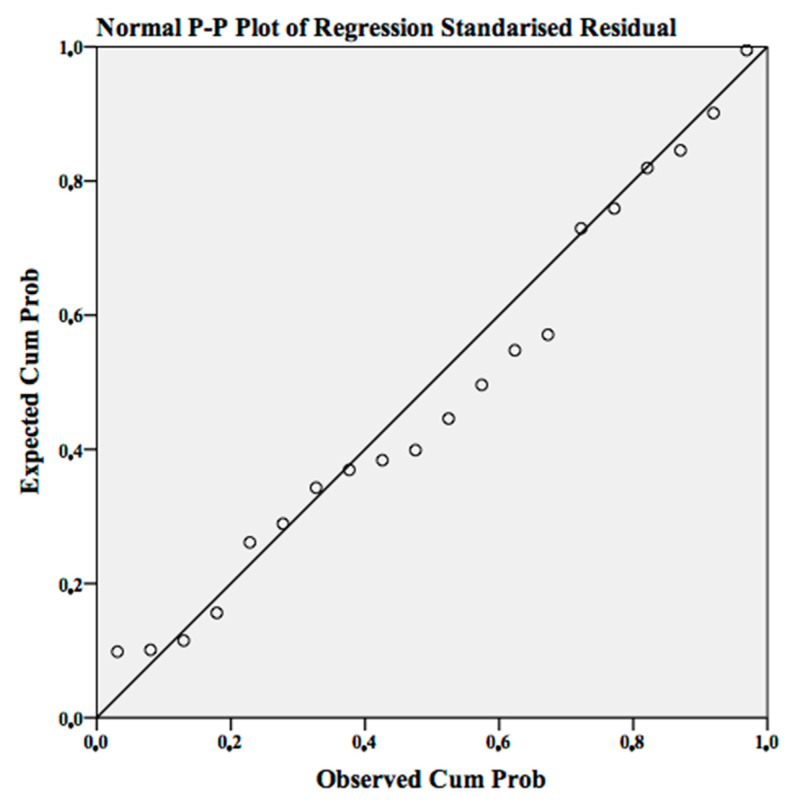
Normal P-P regression graph standardised residue.

The normality of the regression model is fulfilled when the distribution of the standardised residues is normal. In this case, the normality is confirmed in the following normal probability graph (Figure 3), where the points are approximated on the diagonal of the graph.

## 4. Discussion

### 4.1. Incremental Pool Test for Lifeguards (IPTL)

After carrying out the IPTL, it was observed that the VO2 values, in relative terms (44.2 mL·kg·min^−1^), were higher than those obtained in the study by Reilly et al. [19] (36.5 mL·kg·min^−1^) and close to those found by Ruibal-Lista et al. [14] (45.2 mL·kg·min^−1^), both with lifeguards. The values were also close to those found in non-expert swimmers (45.6 mL·kg·min^−1^) in a maximum incremental test [20].

### 4.2. Water Rescue

The average values of maximum heart rate reached during rescue (HR_Rescue_) were around 93% of the maximum heart rate, similar to what happened in previous studies with lifeguards [21], which shows a high energy expenditure and intense cardiovascular demand, as reported by Prieto et al. [10].

From the heart rate reserve, which shows an almost exact relationship with the VO_2max_ [22] it was observed that the average maximum effort during rescue was around 89% of the maximum oxygen uptake reached in the IPTL (VO_2IPTL_). Although the intensity at which a rescue should be performed is not stipulated, some authors argue that it should be below 70% of the lifeguard’s maximum power capacity in order to avoid lactate accumulation [15], thereby reaching a state of metabolic acidosis that could affect other out-of-water activities, such as performing CPR [23]. However, other authors claim that a well-trained lifeguard should be able to perform quality CPR after a demanding rescue (L > 10 mmol·L^−1^) [19].

Independently of the intensity at which the rescue is performed, the application of basic life support in less than 10 min has been shown to be associated with better survival rates in a drowning victim [24]. The less time spent on rescue, the greater the chances of survival [3]. In previous studies, male rescuers completed 150 m rescues without flippers in approximately 260 s [10,25]. Salvador et al. [8] observed that a group of young lifeguards completed a rescue twice the distance, 300 m, in just 288 s, in this case with flippers. In our case, also with flippers, the time for the 200 m rescue was 223 s (3 min and 43 s). This confirms the importance and effectiveness of flippers in the performance of a rescue in natural water spaces [15,26,27].

The data recorded in this study show the possibility that lifeguards with a physical condition and technical mastery similar to those of the lifeguards of our study can perform a rescue of these characteristics in less than 4 min and, although the accumulated fatigue may cause problems when performing other subsequent tasks effectively, we consider that the lifeguard who intervenes in the rescue should do so at maximum intensity, in order to ensure the transfer of the victim to the mainland as soon as possible, and that the lifeguard who has not done the rescue should be the one who should start the cardiopulmonary resuscitation in an effective manner, if necessary [25].

### 4.3. Relationship between IPTL and Water Rescue

The heart rate (HR) values showed differences between the values reached in the IPTL (HRIPTL) and those of the rescue (HRRescue) (*p* < 0.001). It is possible that during the incremental test, the heart rate increases progressively from the beginning of the test until it ends, but in the water rescue the intensity is maximum as of the first moment. It has often been shown that in short, maximum tests, the slow component causes the heart rate and oxygen uptake values not to reach their maximum at the end of the test. However, it has been shown that with short and intense efforts, especially with duration greater than 3 min, it is possible to achieve maximum heart rate and VO_2max_ [28]. Another reason why the maximum HR was not reached could be the intensity applied by the participants, although they were instructed to perform the rescue at maximum intensity from the beginning.

Blood lactate levels were significantly higher in the rescue than in the IPTL (*p* < 0.001). It is possible that increased leg work during the transfer of the victim to the mainland is the reason for these differences [16]. The HR and La values reached, as well as the rescue time (less than 4 min), indicate that anaerobic metabolism is also relevant in the energy contribution during rescue when performed at maximum intensity [25].

The estimated oxygen uptake of the IPTL (VO2IPTL) showed significant correlations with rescue time (R^2^ = 0.37; *p* = 0.05), as Veronese da Costa and his collaborators [29] showed with amateur swimmers in a 400 m freestyle event (R^2^ = 0.55; *p* < 0.05). These results are in line with the recommendations of the United States Lifesaving Association on the importance of developing aerobic power in lifeguards [11].

Recently, Veronese da Costa and his collaborators [20] confirmed with amateur swimmers the correlation between a 400 m freestyle test and the performance of an incremental pool test. In their study, the heart rate, as well as the maximum duration of the test, was measured before and after the test. The authors verified that the maximum time reached in the incremental test correlated significantly with the performance obtained in the 400 m test (R = −0.79; *p* < 0.01). Due to this, they concluded that the performance of this test was related to middle-distance swimming tests, which could also be a tool to design more efficient training and, finally, to evaluate the physical condition of non-expert swimmers.

The results obtained in our study also revealed that the parameter showing the highest correlation with the effectiveness of water rescue is the maximum time reached in the IPTL (Time_IPTL_ y Time_Rescue_; R = −0.77; *p* < 0.001). Based on this correlation, a linear regression model was carried out confirming the relationship between the performance reached in the IPTL and the effectiveness in the rescue (R^2^ = 0.59; *p* < 0.001).

The similarities between the two tests (the swimming style and the material used) made it easier to estimate the effectiveness of the rescue. In the IPTL, as in the first part of the rescue, front crawl swimming was used, a style recommended in a water rescue [30] and with which an energy expenditure similar to that obtained during the transfer of a victim is achieved [31]. At the same time, both tests used flippers, one of the best-known and recommended materials for the performance of the lifeguard’s work [26].

## 5. Conclusions

The maximum oxygen uptake (VO_2ITPL_) and time reached in the IPTL showed a significant correlation with the time spent on the water rescue. The time reached in the IPTL (Time_IPTL_) was shown as the best predictor of the time spent in the rescue (Time_Rescue_) (R^2^ = 0.59; *p* < 0.001). This means that an improvement in IPTL performance could also imply an improvement in water rescue performance.

## Figures and Tables

**Table 1:** Descriptive data on anthropometry and body composition.

Variable	ME	SD	CI (95%)
Age (years)	27.6	7.6	(24.1–31.2)
Height (cm)	175.5	3.3	(174.0–177.1)
Weight (kg)	73.9	7.5	(70.4–77.4)
BMI (kg·m^2^)	24.0	2.3	(22.9–25.1)
%Fat	15.2	2.6	(14.0–16.4)
%Muscle	43.7	2.2	(42.7–44.7)
HR_Rest_ (beats·min^−1^)	65	3	(63–66)

ME: mean; SD: standard deviation; CI: confidence interval.

**Table 2:** Total descriptive data of the results obtained in the pool test (IPTL).

Variable	ME	SD	CI (95%)
VO_2IPTL_ (ml·kg·min^−1^)	44.2	4.7	(42.0–46.4)
HR_IPTL_ (beats·min^−1^)	185	8	(181–189)
La_IPTL_ (mmol·L^−1^)	12.2	2.4	(11.1–13.4)
Time_IPTL_ (s)	726	72	(692–760)

ME: mean; SD: standard deviation; CI: confidence interval.

**Table 3:** Total descriptive data of the results obtained in the rescue test.

Variable	ME	SD	CI (95%)
HR_Rescue_ (beats·min^−1^)	172	9	(168–177)
La_Rescue_ (mmol·L^−1^)	14.0	2.8	(12.7–15.3)
Time_Rescue_ (s)	222	14	(215–229)

ME: mean; SD: standard deviation; CI: confidence interval.

**Table 4:** Analysis of the differences observed between the IPTL and the rescue.

Test/Variable	IPTL			Rescue			Sig. *
	ME	SD	CI	ME	SD	CI	
HR_max_ (beats·min^−1^)	185	8	(181–189)	172	9	(168–177)	<0.001
La (mmol·L^−1^)	12.2	2.4	(11.1–13.4)	14.0	2.8	(12.7–15.3)	0.001

ME: mean; SD: standard deviation; CI: confidence interval; * *T* test for related samples.

**Table 5:** Analysis of the correlations between the variables observed in the IPTL and the effectiveness in the rescue.

Variable	Time_IPTL_	VO_2IPTL_	Time_Rescue_
Time_Rescue_	−0.769 **	−0.607 **	1
	0.000 ^+^	0.005 ^+^	

** Pearson’s correlation (significant at level 0.01); ^+^ Sig. (bilateral).

**Table 6:** Summary of the regression model ^a^.

Model	R	R^2^	R^2^ Adjusted	SE of Estimation	Durbin–Watson
1	0.769	0.591	0.569	9531	1.511

Predictive variables: (constant), maximum time reached in the IPTL (s); ^a^ Dependent variable: time spent on water rescue (s).

**Table 7:** ANOVA of model ^a^.

Model	Sum of Squares	gl	Root Mean Square	F	Sig.
Regression	2365.800	1	2365.800	26.045	0.000
Residual	1635.000	18	90.833		
Total	4000.800	19			

Predictive variables: (constant), maximum time reached in the IPTL (s). ^a^ Dependent variable: time spent on water rescue (s).

**Table 8:** Coefficients of the model ^a^.

Model	Non-Standardised Coefficients		Standardised Coefficients	CI (95%) for B		t	Sig.
	B	Standard Error	Beta	Low. Lim.	Upp. Lim.		
(Constant)	332.983	21.968		286.831	379.136	15.158	0.000
Time IPTL	−0.154	0.030	−0.769	−0.217	−0.090	−5.103	0.000

Predictive variables: (constant), maximum time reached in the Incremental Test in Pool for Lifeguards (IPTL) (Time_IPTL_). ^a^ Dependent variable: time spent on water rescue (Time_Rescue_).
